# The Mechanism of Arsenic Release in Contaminated Paddy Soil with Added Biochar: The Role of Dissolved Organic Matter, Fe, and Bacteria

**DOI:** 10.3390/toxics12090661

**Published:** 2024-09-10

**Authors:** Jianxin Fan, Maoyu Liao, Ting Duan, Ying Hu, Jiaoxia Sun

**Affiliations:** 1 Chongqing Engineering Laboratory of Environmental Hydraulic Engineering, Chongqing Jiaotong University, Chongqing 400074, China; 2 School of River and Ocean Engineering, Chongqing Jiaotong University, Chongqing 400074, China; liaomaoyv@163.com (M.L.); duanting12345@163.com (T.D.); 990020050760@cqjtu.edu.cn (Y.H.); 990201300012@cqjtu.edu.cn (J.S.)

**Keywords:** arsenic, dissolved organic matter, iron, redox potential, bacterial composition

## Abstract

The addition of biochar inevitably modifies the acidity (pH), redox potential (Eh), and dissolved organic matter (DOM) level in the soil. These alterations also have coupled effects on the cycling of iron (Fe) and the composition of bacterial communities, thereby impacting the speciation and availability of arsenic (As) in the soil. This study explored the potential mechanisms through which biochar affects As in paddy soil during flooded cultivation with different pyrolysis temperature biochars (300 °C, 400 °C, and 500 °C) added. The results revealed that the TAs concentration increased in the initial 15 days of soil cultivation with SBC300 or SBC400 addition because increasing the concentration of DOM induced the mobility of As though the formation of As-DOM complexes. Meanwhile, biochar addition elevated the pH, decreased the Eh, and promoted the transformation of specific adsorbed As (A-As) and amorphous iron oxide-bound As (Amo-Fe-As) to supernatant As through enhancing the reductive dissolution of Fe(oxy)(hydr)oxides. Moreover, the biochar altered the relative abundance of As (V)-reducing bacteria (such as *Firmicutes*) and As (III)-oxidizing bacteria (such as *Chloroflex*), thereby affecting As speciation. However, these mechanistic effects varied depending on the pyrolysis temperature of the biochar. The microbial composition of SBC300 and SBC400 were similar, with both containing larger populations of *Enterobacteriaceae* (AsRB) and pseudomonas (FeRB) compared to CK and SBC500. It was proposed that lower pyrolysis temperatures (300 °C and 400 °C) are more favorable for the dissolution of Fe(oxy)(hydr)oxides and the reduction of As (V). However, the biochar from the higher pyrolysis temperature (500 °C) showed environmental impacts akin to the control group (CK). This study demonstrated potential mechanisms of biochar’s effect on As and the role of pyrolysis temperature.

## 1. Introduction

Arsenic (As), recognized as one of the top 10 chemicals of utmost concern for public health by the World Health Organization (WHO), affects countries such as India, Bangladesh, Pakistan, China, the USA, Brazil, and many others [1]. Rice, a crucial staple crop relied upon by over half of the world’s population [2], faces significant risks of As contamination. Rice exhibits a higher susceptibility to As uptake compared to other dryland crops, which is attributed to the anaerobic conditions created by submerged water used in its cultivation, which facilitates As mobilization and biotoxicity [3,4,5]. As is present as inorganic arsenate (As (V)) and arsenite (As (III)) in paddy soil, with As (III) generally considered to be more mobile [6,7]. Therefore, employing cost-effective strategies to reduce the availability of As, particularly inorganic As, in paddy fields contaminated with As is crucial.

Biochar, an efficient adsorbent, has been subjected to extensive research to evaluate its efficacy in remediating As in soil and water environments. Biochar is defined as a material that is produced by pyrolyzing biomass (such as wood, leaves, straw, or manure) at relatively low temperatures (300–700 °C) under oxygen-free or oxygen-limited conditions [8,9]. With a substantial specific surface area, an abundance of surface functional groups (such as -OH, -CH, C=C, and others), and a distinctive pore structure, biochar has demonstrated an exceptional capacity for adsorbing both organic and inorganic molecules [9,10,11]. The properties of biochar are largely influenced by the pyrolysis temperature and feedstock type [12,13,14,15,16]. Specifically, as the pyrolysis temperature increases, the biochar’s pH, carbon content, surface area, pore size, and ash content all increase, while its hydrophilicity, yield, O/C ratio, H/C ratio, and abundance of aliphatic and oxygen-containing functional groups decreases [11,17,18,19]. In summary, biochar pyrolyzed at high temperatures are relatively more effective for As removal, likely due to its high aromaticity, porous structure, and the abundance of mineral phases [20,21]. Biochar can also alter the biochemistry of As in soil; however, few studies have focused on understanding how the pyrolysis temperature influences the biochar’s ability to mediate As through geochemical and biological processes [6,20].

Besides serving as an adsorbent, biochar is a carbon-rich material, implying that its incorporation can elevate the soil organic matter (SOM) concentration and the soil acidity (pH), and buffer the soil redox potential (Eh) and other properties of soil [22,23,24]. Biochar contains a substantial amount of aromatic carbon, which contributes to an increase in the SOM, including sugar, organic acids, and amino acids [25,26]. Additionally, biochar could enhance dissolved organic matter (DOM) release into the soil environment [27,28]. DOM is the most active part of SOM [29], which could directly and indirectly influence the As speciation and availability in paddy fields [6,30,31,32,33,34]. Humic-like DOM could act as electron mediators, enhancing microbial reduction processes and consequently boosting the reductive dissolution of iron (Fe) mineral and As release [35,36,37]. Furthermore, biochar-derived DOM is considered the primary driver of the growth and metabolism of soil bacteria communities, which would impact the soil bacterial diversity, structure, and functions [28,29,38,39,40]. For example, biochar containing more humic-like DOM increased the abundance of *Geobacter* and *Clostridium*, which induced As release [40,41]. However, the properties of the DOM within biochar are predominantly influenced by the feedstock and pyrolysis temperature [30,42,43,44]. Previous studies have indicated that low-temperature biochar has a high DOM content [45,46]. Biochar-derived DOM mainly contains humic-like, fulvic-like, and protein-like substances [43,47]. With increasing pyrolysis temperatures, the relative abundance of humic-like substances first increases and then decreases, and the protein-like substances show the opposite trend [43,48]. This suggests that biochar pyrolyzed at low temperatures might promote As mobility by supplying a higher relative abundance of humic DOM. However, the relationship between microbial and chemistry processes and its effect on As with the incorporation of biochars produced at different pyrolysis temperatures are still unclear. Thus, this study added biochars produced at different temperatures into the soil and investigated the mechanisms of As transformation in the soil.

We hypothesized that the addition of biochars produced at different pyrolysis temperatures might induce differences in paddy soil chemical properties and other soil component changes, including soil pH, soil Eh, DOM, Fe species, and bacterial communities, and indirectly influence the speciation and availability of As. In this study, biochars were prepared at different pyrolysis temperatures and applied to As-contaminated paddy soil, which was then incubated in a short-term flooding system for 21 days. The specific objectives of this work were as follows: (1) to investigate the dynamics of soil pH, Eh, DOM, Fe, bacterial communities, and As after adding biochar; (2) to explore the possible mechanisms through which biochar alters the soil pH, Eh, DOM, Fe, and bacterial communities, thereby influencing As dynamics; and (3) to determine the effect of pyrolysis temperature on these cascade reactions. This study could provide comprehensive insights into the changes in environmental factors when applying biochars produced at different temperatures to As-contaminated paddy soil. This could provide a theoretical foundation for a comprehensive understanding of the mechanisms underlying the biochar-mediated effects on As dynamics. It could also provide new perspectives for the advancement of biochar-based approaches in As pollution management.

## 2. Materials and Methods

### 2.1. Biochar Preparation and Characterization

Three biochar samples were prepared from peanut shells at different temperatures (T = 300 °C, 400 °C, and 500 °C). The reasons for selecting this feedstock and these pyrolysis temperatures are provided in Supplementary Information S1. Under a shield of nitrogen gas, the feedstocks underwent pyrolysis within the controlled environment of a muffle furnace, ensuring a restricted oxygen supply. The heating process was meticulously regulated at a rate of 10 °C per minute, and the target temperature was sustained for 2 h. Subsequently, the biochar samples were gradually cooled to ambient temperature, meticulously ground, and sifted through a 0.15 mm mesh sieve (equivalent to a 100-mesh sieve). The biochar samples were labeled BC300, BC400, and BC500; the characterization methods used for the biochar are described in Supplementary Information S2, and the characterizations results are shown in Supplementary Information S3, Tables S1 and S2, and Figures S1–S5.

### 2.2. Description of Soil Samples

The As-contaminated paddy soil samples for this experiment were collected from Yongzhou City, Qiyang County, Hunan Province, China (26°45′26″ N, 111°52′1″ E). The collected soil samples were dried naturally, sieved through a 10-mesh (2 mm) sieve to remove impurities such as gravel, and plant roots and stems, and bagged. The pH of the As-contaminated soil was 6.74, the concentration of DOM was 22.75 mg/L, and the total arsenic (TAs) content was 93.17 mg/kg. The measurement methods will be described below.

### 2.3. Experiment Design

The soil incubation experiment was conducted over 21 days under laboratory conditions. There were 4 main treatment groups: a control group (CK) and three biochar treatment groups, which included biochar samples prepared at 300 °C (SBC300), 400 °C (SBC400), and 500 °C (SBC500). In each of these biochar treatments, the soil was amended with biochar at a ratio of 5% (*w*/*w*). Within each main treatment group, we further established three subgroups, each corresponding to a different initial soil pH value: 5.5 (P1), 7.0 (P2), and 8.5 (P3). Detailed information is provided in Table S3. A total of 10 g of As-contaminated soil was sieved through a 10-mesh sieve and mixed with 25 mL of deionized water in 50 mL plastic jars and sealed. The jars were randomly divided into 12 groups for the different treatments. Each treatment had 18 replicates, which were placed in light-shielded conditions. To mitigate the introduction of a large amount of oxygen during sampling, each group was evaluated using the destructive testing of 3 randomly selected parallel samples on days 1, 2, 4, 7, 15, and 21. The supernatants from the jars were collected for analysis, including pH, Eh, DOM, Fe, and As analyses. The main detected As and Fe species were As (III), As (V), ferric iron (Fe (III)), and ferrous iron (Fe (II)). We also assessed the As fraction (specific adsorbed As and amorphous iron oxide-bound As) and HCl-extractable Fe (II) (HCl-Fe (II)) in the paddy soil phase. Additionally, the original soil sample and soil samples from the 7th and 21st days were collected for 16s rRNA analysis, focusing on the variation in As (III)-oxidizing bacteria (AsOB), As (V)-reducing bacteria (AsRB), Fe (II)-oxidizing bacteria (FeOB), and Fe (III)-reducing bacteria (FeRB).

### 2.4. The Analysis Methods

#### 2.4.1. Analyses of Supernatant pH, Eh, and DOM Content

The pH and Eh of the soil supernatant were determined using a pH meter and a portable Eh meter (AZ8551, JuChuang Corporation, Shanghai, China). The DOM extraction method was performed as follows. The supernatant samples were filtered through a 0.45 μm filter membrane, and 2–3 drops of HCl were added for preservation. The DOM was analyzed using a total organic carbon (TOC) analyzer (TOC-LCPH/CPN, Shimadzu, Kyoto, Japan) and is represented as the dissolved organic carbon (DOC) content.

#### 2.4.2. The Analysis of Fe and As Levels

The Fe (II) and total Fe (TFe) levels were quantified using the 1,10-phenanthroline spectrophotometry method using UV–visible spectrophotometry (UV-2600, Shimadzu, Japan) [49]. Briefly, the Fe (II) content was analyzed using the 1,10-phenanthroline method. The TFe content was determined using the same procedure except that hydroxylamine hydrochloride was added to transform Fe (III) to Fe (II). The content of Fe (III) was calculated by subtracting the Fe (II) content from the TFe content [50]. The reduced forms of Fe in the paddy soil were extracted using the 0.5 M HCl extraction method [51]. Specifically, 1.0 g of dried soil and 25 mL of 0.5 M HCl were added to a 50 mL centrifuge tube. The mixture was oscillated in the dark at 180 rpm/min for 4 h at 25 °C. After centrifugation, the extract was filtered through a 0.45 μm microporous membrane. The content of HCl-Fe (II) in the soil solid phase was determined using the 1,10-phenanthroline spectrophotometry method.

The total As (TAs) and As (III) contents in the supernatant were measured by atomic fluorescence spectrophotometry (AFS-230E, HaiGuang Corporation, Shanghai, China) [52]. Briefly, the As (III) content was analyzed using the AFS. The TAs content was determined using the same procedure except that HCl and 5% thiourea–ascorbic acid were added to transform As (V) to As (III). The content of As (V) was calculated by subtracting the As (III) content from the TAs content. The determination of the As fractions in the paddy soil was performed using the sequential extraction method [53]. The specific adsorbed As (A-As) and amorphous iron oxide-bound As (Amo-Fe-As) were extracted as follows: (1) 1.0 g of dried soil was accurately weighed and placed in a 50 mL centrifuge tube. A 25 mL volume of 1 M KH_2_PO_4_ and 0.1 M ascorbic acid (pH = 5.0) were added to the tube and oscillated in the dark at 180 rpm/min for 4 h at 25 °C. After centrifugation, the solution was filtered to obtain the extracted As in the form of phosphate, denoted as A-As. (2) To the soil precipitate obtained from the previous centrifugation step, 25 mL of a 0.2 mol/L oxalic acid–ammonium oxalate buffer solution (pH = 3) was added at 25 °C and oscillated in the dark at 180 rpm/min for 4 h at 25 °C. After centrifugation, the solution was filtered to obtain the extracted arsenic in the form of oxalate, denoted as Amo-Fe-As. The content of A-As and Amo-Fe-As in the soil solid phase was determined by atomic fluorescence spectrophotometry.

#### 2.4.3. The 16s rRNA Analysis

The fresh samples and soil samples from the 4th and 21st days were promptly frozen using dry ice and subsequently stored at −80 °C in a freezer. These samples were sent to Shanghai Meiji Biomedical Technology Co. for 16s rRNA sequencing. For each soil sample, the DNA was extracted and assessed by 1% agarose gel electrophoresis. Subsequently, the V4–V5 region of the bacterial 16S rRNA was amplified using specific primers and PCR (ABI GeneAmp 9700) with TransGen AP221-02 and TransStart Fastpfu DNA Polymerase in a 20 μL reaction system. The primers used were 515F (3′-GTGCCAGCMGCCGCGG-5′) and 806R (5′-GGACTACHVGGGTWTCTAAT-3′). After purification of the PCR products, sequencing was performed by Shanghai Meiji Biomedical Technology Corporation (Shanghai, China) using the Illumina MiSeq platform (Illumina, San Diego, USA). The high-throughput sequencing data were analyzed using the QIIME 1.9.1 and Mothur 1.30.2 software packages. Raw sequences were merged into paired-end reads using Flash 1.2.11, followed by clustering of quality-filtered sequences into Operational Taxonomic Units (OTUs) at a 97% sequence similarity threshold using Uparse 11. Each OTU’s representative sequence was taxonomically annotated using the RDP Classifier against the Silva 138.1 database, using a confidence threshold of 70%.

### 2.5. Data Analysis

In the soil incubation section, the results are presented as the mean ± standard error. The statistical analyses were performed using R v4.3.2 (https://www.R-project.org (accessed on 5 September 2024)). The graphs were prepared using the ggplot2 package in R v4.3.2, Origin Pro 2021 (Origin Lab Corp., Northampton, MA, USA), and Adobe Illustrator 2024 (Adobe, San Jose, CA, USA). Before conducting Analysis of Variance (ANOVA) tests, the data underwent screening for normality, homogeneity of variance, and the identification of outliers. Three-way ANOVA (*p* < 0.05) was employed to investigate the effects of pyrolysis temperature, initial soil pH, and incubation time on the soil physicochemical properties as well as the concentrations of different species of Fe and As (Table S4). Chao1, ACE, Shannon, and Simpson diversity indices were calculated to describe the microbial α-diversity (Table S5), and the bacteria communities under different treatments were analyzed using a cluster analysis. Redundancy Analysis (RDA) was conducted to assess the impact of pH, Eh, DOM, and Fe and As levels on the bacterial communities. The RDA was performed using the vegan package in R. Pearson’s (two-tailed) correlation analysis was used to analyze correlations among different variables via the corrplot package in R. The Mantel test was used to test the correlation between the distance matrix of environmental variables and the As matrix via the ggcor package in R.

## 3. Results

### 3.1. pH, Eh, and DOM Dynamics in the Supernatant

The dynamics of supernatant pH, Eh, and DOM under the different treatments are shown in Figure S6. From day 1 to 21, the pH values of the groups supplemented with biochar were consistently higher than those of the CK group (Figure S6a). SBC500-P1, SBC500-P2, and SBC400-P3 showed the greatest increase in pH, raising it by 0.4, 0.32, and 0.42 units, respectively, on day 21 from different initial soil pHs. The trend in pH changes for the CK group showed a gradual increase, approaching neutrality by day 7. Conversely, in the samples supplemented with biochar, the pH levels exhibited an initial rise followed by a decline, reaching their maximum values on day 15. The Eh values of the SBC300 and SBC400 groups were lower than that of the CK group, but SBC500 exhibited a higher Eh (Figure S6b). As shown in Figure S6c, the impact of the biochar addition on the changes in DOM in the supernatant varied depending on the initial pH level. During the incubation, the addition of biochar significantly increased the DOM content in the supernatant. The DOM content increased and then decreased, with the peak occurring on day 4 or day 7. When the initial soil pH was 8.5, each experimental group experienced the most significant increase in DOM, with the DOM content of SBC300-P3, SBC400-P3, and SBC500-P3 increasing by 42.5%, 36.6%, and 22.7%, respectively.

### 3.2. Fe and As Dynamics

#### 3.2.1. Fe and As Dynamics in Supernatant

**Figure 1:**
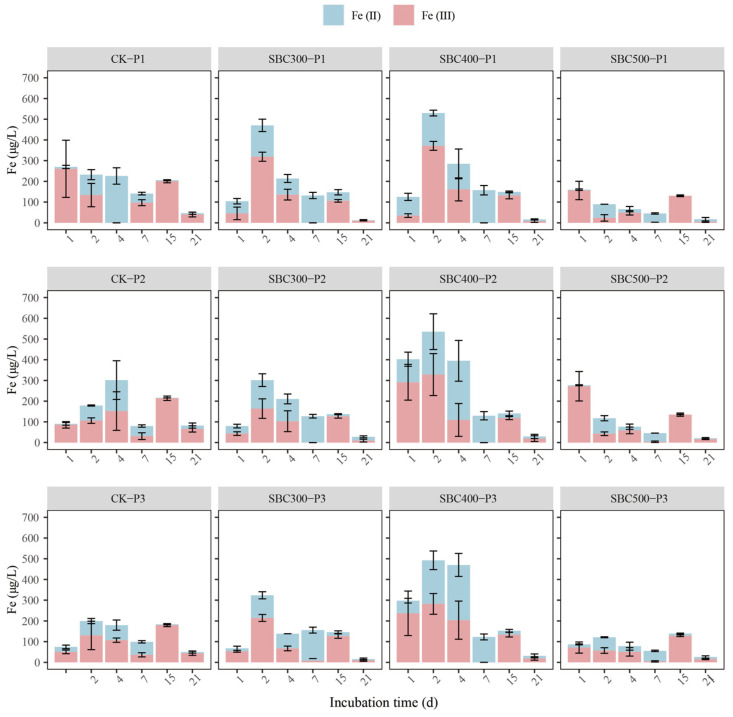
Concentrations of Fe in the supernatant with different treatments during the incubation. Values are the mean ± standard error of three replicates. Different letters on the same day indicate significant differences among treatments (*p* < 0.05). Treatment abbreviations are defined in Table S3.

The supernatant Fe concentration differed among the 12 treatments during the incubation (Figure 1). During the initial 4 days of incubation, the TFe concentration in SBC300 and SBC400 was higher than that of CK and SBC500. The TFe dynamics in SBC300 and SBC400 exhibited an initial rise followed by a decline, reaching their maximum values on day 2 or 4. Conversely, the variation in the Fe concentration of SBC500 and CK showed an ‘M’-shaped pattern. Compared to CK, the TFe concentration in the supernatant was significantly reduced in SBC300, SBC400, and SBC500 after 21 days of incubation. However, the differences among SBC300, SBC400, and SBC500 were not pronounced, and the reduction in the TFe content was approximately 70%. Fe (II) remained dominant throughout the incubation period except for day 15. On the contrary, Fe (III) was dominant only on day 15.

**Figure 2:**
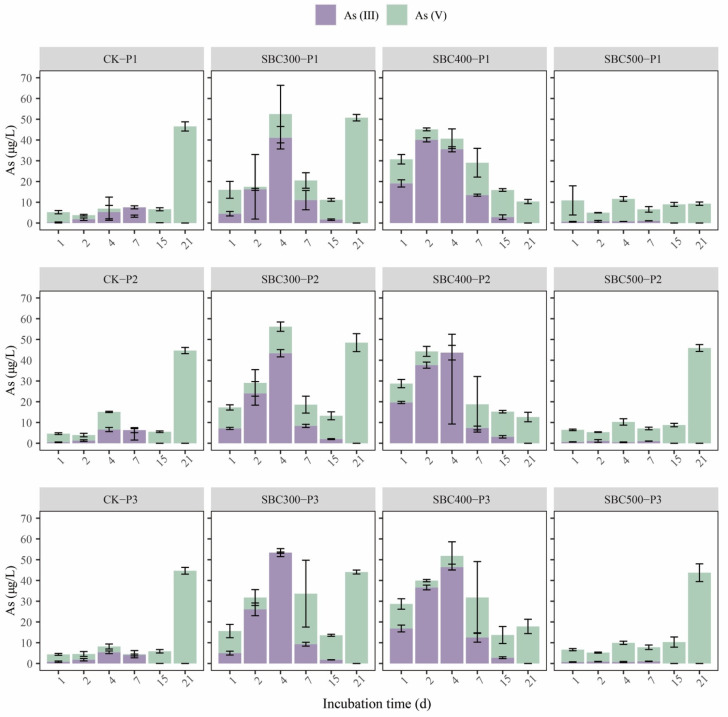
Concentrations of As in the supernatant with different treatments during the incubation. Values are the mean ± standard error of three replicates. Treatment abbreviations are defined in Table S3.

The variations in the TAs content were similar to those for the TFe content mentioned above (Figure 2). During the first four days of incubation, the supernatant TAs concentration in SBC300 and SBC400 was higher than in the CK group, while the difference between SBC500 and CK was not substantial. From day 1 to day 15, the TAs concentrations in SBC300 and SBC400 both showed an initial increase followed by a decrease, reaching their maximum values on day 2 or day 4. On day 21, SBC400 demonstrated a notable reduction in the TAs content, reducing the TAs concentration in the supernatant by 77.7%, 71.7%, and 60.0% under the three different initial soil pH conditions, respectively. The variation in TAs content between SBC500 and CK was similar, remaining stable in the first 15 days and experiencing an increase by day 21. SBC500-P1 showed a significant removal of As. Throughout the entire experiment, As (V) predominantly dominated in both CK and SBC500. The CK group on day 4 and day 7 showed a slight increase in As (III). In contrast, for SBC400 and SBC500, the proportion of As (III) rapidly increased from day 1 to 4, followed by a gradual decrease to non-detectable levels from day 7 to 21.

#### 3.2.2. Fe and As Dynamics in Soil

The concentration of HCl-Fe (II) remained stable during the first 4 days and then steadily increased, and the groups with the addition of biochar had an elevated concentration on day 21 (Figure S7). Furthermore, when the initial soil pH was 7.0 or 8.5, the effects of the SBC300 and SBC400 treatments were similar, differing from the treatment effect of SBC500. The addition of the three biochars uniformly increased the content of HCl-Fe (II) in the soil by approximately 135%. When the initial soil pH was 5.5, SBC500 exhibited the highest increase, showing a remarkable growth of 147.47%. However, when the initial soil pH was 7.0 or 8.5, SBC400 produced the most significant enhancement, with growth rates of 134.25% and 136.44%, respectively.

**Figure 3:**
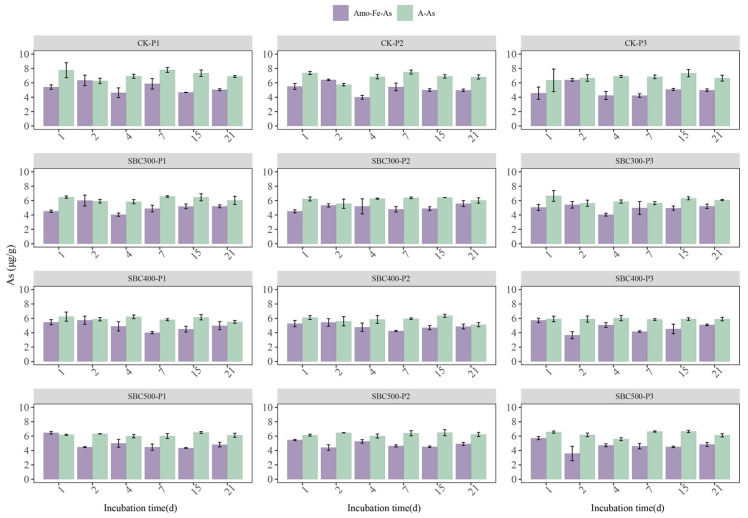
Concentrations of As fractions in the paddy soil with different treatments during the incubation. Values are the mean ± standard error of three replicates. Treatment abbreviations are defined in Table S3.

The addition of biochar had a significant impact on both the A-As and Amo-Fe-As concentrations (Figure 3). On day 1, the concentrations of A-As and Amo-Fe-As in SBC300 and SBC400 were significantly lower than those in the CK group, while those of SBC500 were similar to those of CK. The overall trend of the A-As concentration showed a decrease from day 1 to day 2, followed by an increase from day 2 to day 4, stabilizing by day 7. This trend was more pronounced when the initial pH was 5.5 or 7.0, and the addition of biochar enhanced the decrease in the A-As level during the first 2 days and mitigated the subsequent increase in the A-As level. In the CK group, the overall variation in the Amo-Fe-As concentration exhibited an ‘M’-shaped pattern, reaching its maximum values on day 2 and day 7 or 15. However, SBC300 and SBC400 showed similar changes to the CK group in the first 7 days, but after day 7, the content of Amo-Fe-As continued to increase, particularly in SBC400. SBC500 exhibited a trend of first decrease and then increase, stabilizing after day 4. After a 21-day cultivation period, there was a noticeable decrease in the A-As concentration in SBC300, SBC400, and SBC500, although there were no discernible differences among these groups. Additionally, the content of Amo-Fe-As increased, but there were no differences among the groups.

### 3.3. Dynamics of Bacterial Diversity and Composition

The addition of biochar influenced the microbial parameters (OTUs, ACE, Chao1, Simpson, and Shannon indices) in the rice paddy soil (Table S7). On day 7, the OTUs in all the experimental groups and CK were lower than those in the original soil, and in the treatments with added biochar, the numbers of OTUs were lower than that of CK. Compared to day 4, the number of OTUs in the soil decreased at day 21 in CK. However, the addition of biochar notably mitigated the decline in OTUs, with particularly noteworthy increases observed for SBC300-P3, SBC400-P1, and SBC400-P2, leading to increases of 10.43%, 6.78%, and 5.60%, respectively. Indices such as ACE and Chao1, reflecting the unobserved species richness in the soil, exhibited patterns of change consistent with that of the OTUs. Additionally, the Simpson and Shannon indices displayed the same trends, both indicating increased diversity on day 21 compared to day 7.

#### 3.3.1. Bacterial Composition Dynamics at the Phylum Level

**Figure 4:**
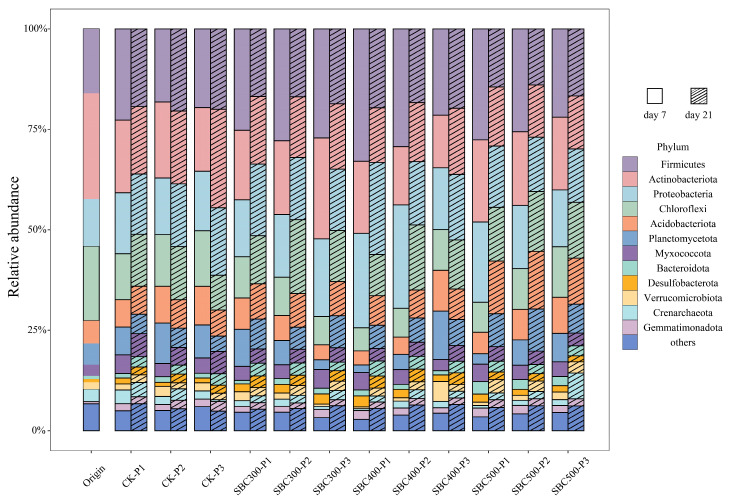
Relative abundances in bacterial community composition among the different amendment treatments at the phylum level. The bar color represents the bacterial phylum. Origin is the fresh soil without any treatment. Treatment abbreviations are defined in Table S3.

The bacterial community structure varied between the soils with different treatments (Figure 4). In the fresh soil samples, the predominant bacterial phyla were *Firmicutes*, *Actinobacteriota*, *Proteobacteria*, *Chloroflexi*, *Acidobacteriota*, and *Planctomycetota*, constituting 16.02%, 26.31%, 11.72%, 18.57%, 5.66%, and 5.29% of the total, respectively. On day 7, under all three initial soil pH conditions, the relative abundances of Firmicutes in SBC300, SBC400, and SBC500 were higher than in CK; however, by day 21, the abundance declined to values lower than those in CK. Conversely, the *Chloroflexi* dynamics showed the opposite trend to that of Firmicutes. Apart from SBC300, where the abundance of *Actinobacteriota* did not show a significant difference compared to CK, it notably decreased in the other groups on day 21. When the initial soil pH was 5.5, SBC400-P1 exhibited the maximum decrease of 19.17%. When the initial soil pH was 7.0 or 8.5, SBC500-P2 and SBC500-P3 experienced the most substantial reductions, decreasing by 28.31% and 46.26%, respectively. When the initial soil pH was 5.5, the addition of biochar promoted an increase in the abundance of Proteobacteria. When the initial soil pH was 7.0 or 8.5, biochar addition led to a decrease in their abundance. Regardless of the initial soil pH, the addition of biochar enhanced the relative abundances of *Acidobacteriota* and *Planctomycetota* on day 21, with SBC500 exhibiting the most favorable effects.

#### 3.3.2. Bacterial Composition Dynamics at the Genera Level

*Bacillus*, *norank_f__67-14*, *norank_f__Anaerolineaceae*, *norank_f__norank_o__Gaiellales*, and *Gaiella* occupied prominent positions among the genera observed (Figure S8). In the original soil samples, the top 10 genera exhibited a relatively even distribution. However, following the flooding cultivation, the relative abundance of Bacillus noticeably increased, and the groups with the addition of biochar had a significantly higher abundance. By day 21, the relative abundance of the major genera generally declined compared to day 7 across all groups. During this experiment, certain bacterial groups were identified (Supplementary Figure S9). Among the AsRB, Stenotrophomonas and Bacillus were observed. Within the AsOB, Bacillus, Vibrio, Enterobacteriaceae, and Pseudomonas were detected. For FeRB, the presence of Pseudomonas and Thiobacillus was noted. There was no evidence for the presence of FeOB.

#### 3.3.3. The Results of Cluster Analysis and RDA

**Figure 5:**
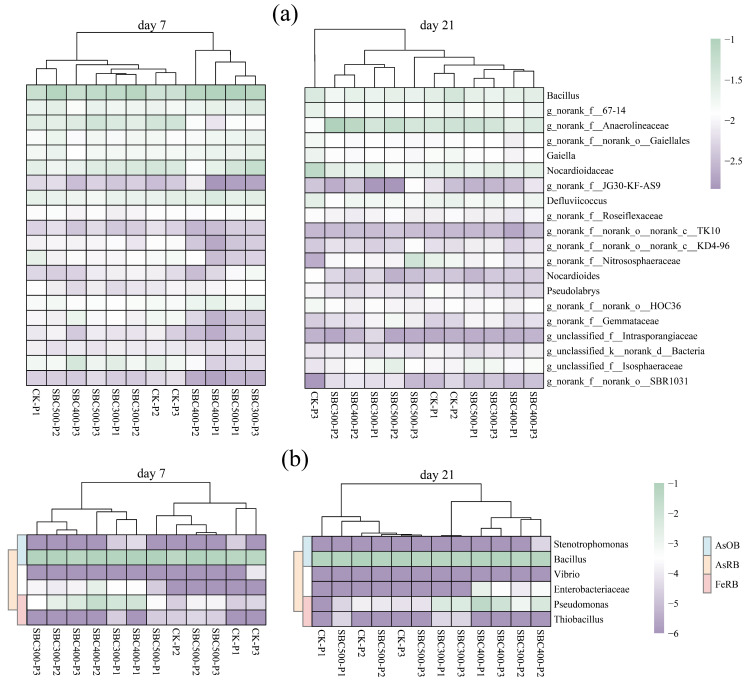
Clustering heatmap based on relative abundances of the top 20 genera (>1% each) (**a**) and heatmap of relative abundances of the main AsOB, AsRB, and FeRB in the experiment (**b**). Cluster analysis was performed based on the Euclidean distance classification method. All relative abundance data have been logarithmically transformed for amplification: (**a**) was amplified using the logarithmic function lg(x), while (**b**) was amplified using the logarithmic function lg(x + 0.000001) where x is the relative abundance of the genus. Treatment abbreviations are defined in Table S3.

The results of the cluster analysis indicated that the different biochar treatments, along with the initial soil pH treatments, affected the structure of the soil bacteria community at the genus level (Figure 5a). By day 7, the soil bacterial communities had predominantly segregated into two distinct clusters: one composed of samples subjected to the SBC400-P1, SBC400-P2, SBC500-P1, and SBC300-P3 treatments, and the other comprising the remaining experimental treatments. By day 21, the differences between the various treatments were no longer significant. However, the disparities among the treatments regarding the AsOB, AsRB, and FeRB were evident (Figure 5b). On both day 4 and day 21, the CK and SBC500 groups exhibited similar effects, while the SBC300 and SBC400 groups demonstrated comparable outcomes. SBC300 and SBC400 promoted the proliferation of Enterobacteriaceae and Pseudomonas, fostering their growth and reproduction.

**Figure 6:**
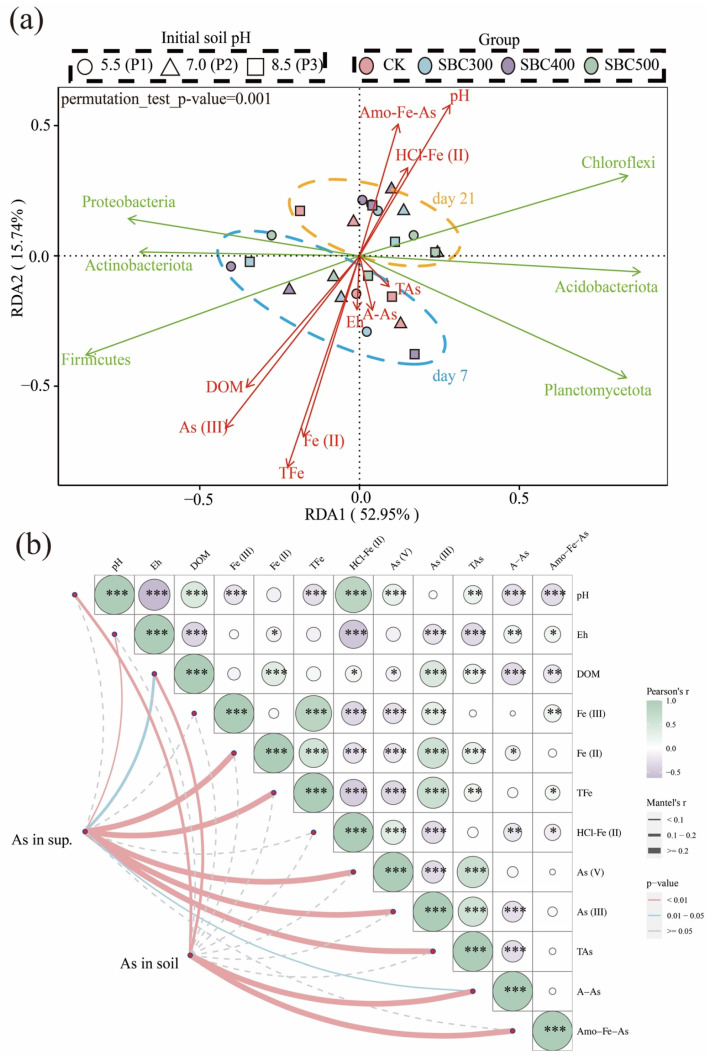
(**a**) Results of Redundancy Analysis (RDA) at the phylum level. The biological and environmental matrices encompass data from day 4 and day 21. RDA was performed to investigate the impacts of environmental factor changes on the bacterial community. Considering the collinearity between TAs and As (V) levels, as well as TFe and Fe (III) levels, (**b**) uses TAs and TFe levels as the environmental factors. The red arrows indicate environmental factors, and the green arrows indicate the main bacteria of that phylum. Treatment abbreviations are defined in Table S3. (**b**) Mantel test-based correlations between environmental factors and As levels with parameters. Mantel’s r value is represented by the edge width, and statistical significance is represented by the color. Pair-wise Pearson’s coefficients between parameters are shown as a color gradient. Significant differences are labeled with * *p* < 0.05, ** *p* < 0.01, and *** *p* < 0.001. As in sup. refers to the As concentration in the supernatant.

The bacterial community was strongly affected by the changing soil environmental factors, as revealed by the permutation test (*p* = 0.001) (Figure 6a). The compositions of the community on day 7 and day 21 displayed a significant difference, but there were only moderate differences between the different treatments. Additionally, the differences between the treatments on day 21 were smaller compared to those on day 7. The variation in bacteria species showed a strong response to changes in the DOM, pH, and TFe, Fe (II), As (III), and Amo-Fe-As levels. *Firmicutes*, *Actinobacteriota*, *Acidobacteriota*, and *Planctomycetota* exhibited a positive relationship with the DOM, Fe, Fe (II), and As (III) levels, but a negative relationship with the Amo-Fe-As HCl-Fe (II) levels and pH. On the other hand, *Proteobacteria* and *Chloroflexi* displayed contrasting patterns in their relationships with these variables.

### 3.4. The Results of the Mantel Test and Pearson Correlation Analysis

The Mantel test was used to evaluate the effects of the environmental factors on the soil and supernatant As concentrations (Figure 6b). Among all the parameters tested, the supernatant As concentration was significantly related to the Eh, supernatant Fe (II), TFe, A-As levels (*p* < 0.01), and DOM (0.01 < *p* < 0.05), but the soil As concentration was only significantly related to DOM and pH (*p* < 0.01). This suggests that the DOM content is the most important factor affecting the mobility of As. Furthermore, the Pearson correlation analysis showed that the TAs level was positively and significantly correlated to the DOM, TFe, Fe (II), and HCl-Fe (II) levels, but negatively and significantly related to the Eh and A-As concentration. As (V) was both significantly and negatively related to the TFe, Fe (II), and Fe (III) levels, and positively correlated with HCl-Fe (II) levels. The As (III) content showed the opposite relationship to those of As (V). The A-As and Amo-Fe-As levels exhibited significant and negative correlations with pH and DOM, but a positive correlation with Eh.

## 4. Discussion

### 4.1. Effect of Pyrolysis Temperature on Soil Component Dynamics

The increase in the supernatant pH in SBC300, SBC400, and SBC500 suggested that the addition of biochar significantly elevated the soil pH, which is consistent with observations from previous studies [54,55,56]. The increase in pH was mainly caused by the alkaline groups (such as -COO- and -O-) and ash in the biochar (Table S1) [57], while the decrease in pH on day 15 might have resulted from the dissolution of inorganic or soluble alkalis presented on the surface of the biochar [22]. However, the pyrolysis temperature had a minimal impact on the supernatant pH in this study. The variation in Eh among the different groups suggested that biochar has a reducing capacity when prepared at low temperatures and is converted to an oxidizable form at high temperatures, consistent with that results from previous studies [31,58].

The difference in the DOM concentration in the supernatant among the different pyrolysis temperature groups was consistent with the differences in relative abundance of DOM in SBC300, SBC400, and SBC500, indicating a decrease in DOM with increasing pyrolysis temperature. Generally, it was observed that the addition of biochar increased the soil pH and DOM but decreased the soil Eh. Focusing on the pH, Eh, and DOM dynamics, the approximate turning point between days 7 and 15 suggested that the soil modification capability of biochar might vary with its aging process, and that biochar aging occurs relatively quickly.

In this study, it was found that the introduction of biochar appears to increase the microbial diversity in flooded paddy soil. In this study, the dominant bacterial phyla were *Firmicutes*, *Actinobacteriota*, *Proteobacteria*, *Chloroflexi*, *Acidobacteriota*, and *Planctomycetota*, which is similar to the results of previous research in As-contaminated environments [59,60,61]. *Firmicutes* was identified as a dissimilatory As (Ⅴ)-reducing prokaryote [62,63]. Conversely, *Chloroflexi* and Proteobacteria are potential As (III)-oxidizing bacteria in As-bearing geothermal environments [63,64]. Furthermore, *Actinobacteriota* is an important taxa for reducing heavy metal (loid) mobility [61]. Integrating these results with our RDA analysis results, we suggested that *Chloroflex* and Firmicutes were the main bacteria phyla influenced by the addition of biochar, which then increased As speciation and availability. On day 7, the addition of biochar increased the abundance of *Firmicutes*, with SBC400 showing the greatest increase, which was particularly evident when the initial soil pH was 5.5 or 7.0. However, on day 21, the groups with the addition of biochar had a decreased *Firmicutes* abundance. The dynamics of *Chloroflex* were opposite to those of Firmicutes. It is interesting to compare day 7 with day 21, as it shows that the effect of biochar on bacteria varies over time for Firmicutes and *Chloroflex*.

At the genus level, it was observed that biochars prepared at lower pyrolysis temperatures were beneficial for certain FeRB and AsRB, such as Pseudomonas (Figure 5b). However, clear evidence of effects on FeOB was not observed, nor was the presence of betaproteobacteria associated with Fe (III) reduction detected in any of the groups [65,66]. The cluster analysis showed that the CK and SBC500 groups exhibited similar effects, while the SBC300 and SBC400 groups demonstrated comparable outcomes on days 7 and 21. The results of the RDA indicated that both biochar and the initial pH of the soil have a notable impact on bacterial communities at the phylum level. Significant differences were found among all the groups, and these differences were observed to gradually decrease as the cultivation progressed. These suggest that the pyrolysis temperature at which the biochar is produced might influence the soil bacterial community structure. The low-temperature biochar promoted the increase in abundance of *Enterobacteriaceae* (AsRB) and pseudomonas (FeRB), while the high-temperature biochar had a slight effect on the community structure. The effect of biochar on the community also diminished as the biochar aged. These effects might be attributed to the pyrolysis temperature affecting the biochar properties, such as pH, SOC, and porosity, which strongly affect microorganisms [67,68,69,70].

SBC300 and SBC400 showed a higher increase in TFe, Fe (II), and Fe (III) concentrations on day 2. This indicates that biochars prepared at low temperatures have the ability to promote the reduction of Fe (III) and the reductive dissolution of Fe (oxy) (hydro) oxides. A possible explanation for this might be that the DOM acts as electron donors to mediate the electron transfer processes which induces Fe (III) reduction [71]. The peak of this reduction process was reached on day 7, after which, the Fe (II) rapidly diminished, accompanied by a sharp decrease in Fe (III). Additionally, starting from day 7, the differences among the various treatments diminished rapidly, which was consistent with the pH, Eh, and DOM dynamics. The reduction in the TFe concentration in the supernatant on day 21 indicated that the application of biochar led to an increased accumulation of Fe in the soil phase. The exchangeable Fe content in the soil could serve as the recipient of the supernatant Fe [72]. Thus, it was inferred that the dissolution of Fe (oxy) (hydro) oxides occurs within the first 4 days, followed by regeneration.

In summary, the addition of biochar indeed influences the soil pH, Eh, DOM, Fe content, and bacterial community structure, while different pyrolysis temperatures led to variations in these factors. These variations were advantageous for exploring the mechanism of biochar’s effects on As dynamics, as well as the differences in mechanisms among the biochars produced at different pyrolysis temperatures.

### 4.2. Possible Mechanisms for Biochar’s Effects on As Dynamics

Combining the results of the RDA, Mantel test, and Pearson’s correlation analysis, it was observed that DOM was the most significant influencing factor. Additionally, the Eh, pH, cycling of Fe, and the dynamics of *Firmicutes* and *Chloroflex* also impacted As speciation and availability, but their effects on As dynamics were linked to changes in DOM, as was observed in previous studies [32,73,74,75].

As depicted in Figure 6a, a positive correlation was observed between the As (III) content and Firmicutes abundance, while a negative correlation was found with the *Chloroflex* abundance. This phenomenon is consistent with their role in the cycling of As. Although Proteobacteria and Actinobacteriota have been demonstrated to affect As and Fe cycling, the results of the RDA did not show a significant correlation. Thus, we inferred that *Firmicutes* and *Chloroflex* were the main bacteria affected by the biochar and subsequently influenced As speciation.

As shown in Figure 6b, the results of the Mantel test indicated that only DOM was significantly related to As levels in both the supernatant and the soil. These findings were attributed to the ability of DOM to complex As in the soil (DOM-As). However, DOM also possesses a strong migration capacity, thereby enhancing the mobility of As [74,76]. Furthermore, DOM could compete with As for adsorption sites on Fe(oxy)(hydr)oxides, resulting in the release of As into the aqueous phase [35,77]. The positive correlation between DOM and the supernatant TAs level, as well as the negative correlation between DOM and the soil As level (A-As and Amo-Fe-As), also supports this potential mechanism.

A Pearson’s correlation analysis was conducted to examine the relationships among all the soil factors. Focusing on the TAs, A-As, and Amo-Fe-As levels, we explored the mechanisms through which biochar influences As availability (migration between aqueous and solid phases). pH was negatively and significantly correlated with both A-As (*p* < 0.01) and Amo-Fe-As (*p* < 0.001) levels, which is consistent with the results of a previous study that indicated that higher pH values enhance the release of As [78]. It is difficult to explain this result, but it might be related to (1) the increase in pH resulting in the release of As due to the lower stability of otherwise stable metal oxide As complexes [79,80], and (2) the higher pH (6-8) can generate the most mobile As (III) species H_3_AsO_3_ [33,81]. Eh was positively correlated with the TAs level (*p* < 0.01) but negatively correlated with the A-As level (*p* < 0.05). This may be attributed to the reduction in the dissolution of Fe(oxy)(hydr)oxides, releasing As. The positive correlation between the TAs and TFe levels (*p* < 0.01) also indicated that the dissolution of Fe(oxy)(hydr)oxides is a potential factor affecting As mobility.

In summary, the addition of biochar (1) increased the DOM concentration leading to the mobility of As; (2) increased the pH, enhancing As mobility; (3) decreased the Eh and promoted the transformation of A-As and Amo-Fe-As to supernatant As; and (4) altered the relative abundance of Firmicutes and Chloroflex, thereby affected As speciation.

### 4.3. Effect of Pyrolysis Temperature on As Transformation

In this section, the conclusions of Section 4.1 and Section 4.2 are combined to explain the dynamics of As speciation under the different treatments and to explore the effect of pyrolysis temperature on the possible mechanisms. As shown in Figure 1 and Figure 2, it was evident that the effects of biochar on As dynamics could be divided into two stages, with the first stage occurring from day 1 to day 4, and the second stage from day 7 to day 21. It is speculated that this was not only related to the addition of biochar but also to the changes in redox conditions during the early stages of flooded cultivation based on the immediate decline in Eh (Figure S6b) and the results from previous studies [82,83].

At the beginning of the incubation period (from day 1 to day 4), SBC300 and SBC400 showed a higher increase in the supernatant TAs concentration, especially the As (III) concentration. However, there was no increase in supernatant TAs and As (III) levels in SBC500 (Figure 2). The concentration of A-As decreased in SBC300, SBC400, and SBC500 during the first 4 days (Figure 3). This suggested that a low pyrolysis temperature is beneficial for biochar’s ability to immediately release As. This was related to the higher content of DOM in the biochars prepared at lower pyrolysis temperatures promoting the release of As. During this period, the main species formed was As (III), likely resulting from reduction reactions during the initial flooding period and the increase in Firmicutes and decline of *Chloroflex* on day 4 due to the addition of the biochar. Furthermore, the SBC300 and SBC400 groups in this experiment showed higher reducibility, leading to the transformation of As (V) to As (III) and the reductive dissolution of Amo-Fe-As (Figure 3). However, throughout the entire incubation process, the As in SBC500 remained predominantly in the form of As (V). The predominance of As (V) was attributed to the distinct composition of FeRB and AsRB in SBC500 compared to SBC300 and SBC400 (Figure 4 and Figure 5b).

During the second stage, the proportion of As (III) decreased until it reached undetectable levels (Figure 2). This indicated that after the stabilization of flooding in this experiment, the As primarily existed in the form of As (V), significantly reducing its mobility. This was related to the decline in Firmicutes and the increase in *Chloroflex* on day 21. On day 21, only the TAs content in the supernatant of SBC400 decreased among the three experimental groups with biochar addition. Furthermore, the increase in Amo-Fe-As and HCl-Fe (II) in SBC400 (Figure 3 and Figure S7) indicated the regeneration of Fe(oxy)(hydr)oxides in this stage and their recombination with As. This was related to the formation of more stable ternary compounds (As-Fe-DOM), as suggested in previous studies, where amorphous Fe oxides bind with As and DOM [31,84]. However, an increase in HCl-Fe (II) and Amo-Fe-As was also observed in SBC300 (Figure 3 and Figure S7), without a decrease in the TAs content (Figure 2). It was speculated that this was due to the competition for complexation between DOM and As, as well as the formation of DOM-As [85,86]. BC300 had a higher DOM content (Table S2), leading to the possible formation of DOM-Fe-DOM rather than As-Fe-DOM, and the higher DOM content also implies more DOM-As, all of which increase the mobility of As. Surprisingly, SBC500-P1 was found to reduce the supernatant TAs content on day 21 as well. Explaining this result is challenging, but it might be associated with the lower initial pH favoring the adsorption of As by soil components [87,88], thus preventing As release by day 21.

**Figure 7:**
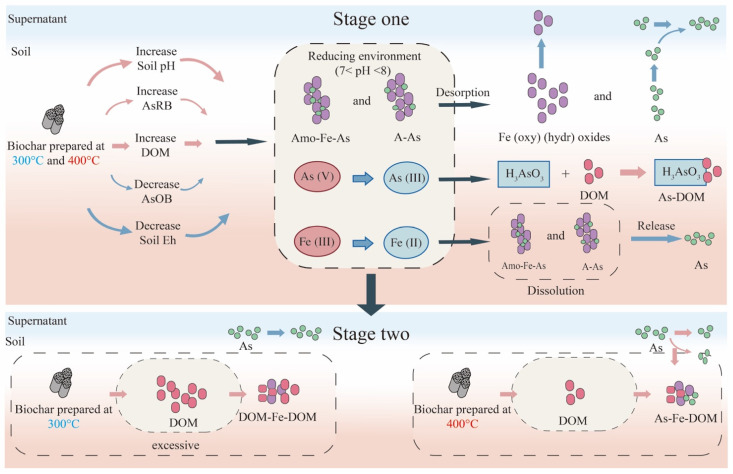
Possible mechanisms of biochar’s effects on arsenic in rice paddy soil under different treatments.

In summary, this study illustrated the potential mechanisms by which biochar affects the speciation and mobility of As, along with the impact of pyrolysis temperature on these mechanisms (Figure 7). The use of lower pyrolysis temperatures (300 °C and 400 °C) to produce the biochars promoted the transfer of As to the aqueous phase during the initial cultivation stage by (1) promoting the reductive dissolution of Fe(oxy)(hydr)oxides through an increase in pH and a decrease in Eh, (2) converting As (V) to As (III) by increasing *Firmicute* populations and decreasing *Chloroflex* populations, and (3) enhancing the concentration of DOM to form soluble As (As-DOM) and compete for adsorption sites on minerals. However, during the subsequent cultivation, the biochars produced at 400 °C formed stable ternary complexes with regenerated Fe(oxy)(hydr)oxides and DOM, resulting in a decrease in As levels in the supernatant. In contrast, biochars produced at 300 °C, due to their excessively high DOM content, interfered with the binding of As to Fe(oxy)(hydr)oxides; thus, there was no decrease in As levels in the supernatant. Therefore, it is suggested that low-temperature pyrolysis biochar should not be applied to As-contaminated soils unless it has been modified.

## 5. Conclusions

Biochars prepared at different pyrolysis temperatures were added into flooded paddy soil with varying initial pH values; it was observed that the impact of the biochar on the soil properties and As species was far more pronounced than that of the initial soil pH. Higher pyrolysis temperatures favored an increase in pH and Eh but led to a decrease in DOM. Additionally, the addition of biochar significantly reduced the TFe concentration in the supernatant, while increasing the concentration of HCl-Fe (II) in the soil. Biochars produced at lower pyrolysis temperatures promoted AsOB, AsRB, and FeRB in the soil. However, in short-term experiments, DOM demonstrated the greatest influence on As behavior. This study illustrated the potential mechanisms through which biochar affects the speciation and mobility of As, along with the impact of pyrolysis temperature on these mechanisms. By doing so, it has made a significant contribution to understanding the mechanisms underlying the influence of biochar on As migration. Furthermore, it provides new perspectives for addressing environmental issues related to the remediation of As contamination using biochar. However, there further experiments are needed to validate and enhance our conclusions. For instance, future studies should determine (1) whether these mechanisms are affected by the feedstock of the biochar; (2) whether these patterns also exist in longer-term flooding experiments or under conditions of wet–dry cycles; (3) whether utilizing metagenomic sequencing instead of 16S rRNA can better depict the role of microbial functional genes in the mechanisms; and (4) whether the soil-to-supernatant ratio used in this experiment, while amplifying the conversion between liquid and solid phases, also leads to the oxidation of elements in the supernatant, thus affecting the valence states of As and Fe.

## Data Availability

Data are available upon reasonable request to the corresponding author.

## Supplementary Materials

The following supporting information can be downloaded at: https://www.mdpi.com/article/10.3390/toxics12090661/s1, S1: Reasons for choosing the selected feedstock and pyrolysis temperatures; S2: Biochar characterization methods; S3: The results and discussion of the biochar characterization; Table S1: The pH and variation in DOM quality indices of biochars; Table S2: Secondary structures in different fouling layers; Table S3: The experiment group in the soil incubation experiment; Table S4: The three-way Analysis of Variance (ANOVA) (*p* < 0.05) results for the effects of different treatments on incubated soil (* *p* < 0.05; ** *p* < 0.01, *** *p* < 0.001); Table S5: Alpha-diversity indices of bacterial communities in soil samples under the different treatments; Figure S1: Scanning electron microscope (SEM) images of biochar samples; Figure S2: The FTIR spectra of different biochar samples produced at different pyrolysis temperatures; Figure S3: The UV–Vis spectra of DOM derived from biochars produced at different pyrolysis temperatures; Figure S4: The characteristic parameters of the UV–Vis spectra of the DOM released from the biochars produced at different pyrolysis temperatures; Figure S5: The EEM of DOM derived from biochars produced at different pyrolysis temperatures; Figure S6: Concentrations of Fe in the soil phase with different treatments during the incubation; Figure S7: Relative abundances in bacterial community composition among the different amendment treatments at the genus level; Figure S8: Relative abundances of main AsOB, AsRB, and FeRB among the different amendment treatments; Figure S9: Relative abundances of main AsOB, AsRB, and FeRB among the different amendment treatments. Refs. [27,43,57,89,90,91,92,93,94,95,96,97,98,99,100,101,102,103,104,105] are cited within Supplementary Materials.
