# Supplementary material for: The Mechanism of Arsenic Release in Contaminated Paddy Soil with Added Biochar: The Role of Dissolved Organic Matter, Fe, and Bacteria
Source: Toxics. 2024 Sep 10;12(9):661. doi: 10.3390/toxics12090661 (PMC11435835; doi:10.3390/toxics12090661)
Supplement: Supplementary file 1 [file toxics-12-00661-s001.zip › toxics-3176652-supplementary.pdf]

Supplementary Materials for

The Mechanism of Arsenic Release in Contaminated Paddy Soil with Added Biochar: The Role of Dissolved Organic Matter, Fe, and Bacteria

Jianxin Fan*[1,2], Maoyu Liao[2], Ting Duan[2], Ying Hu[2], Jiaoxia Sun[2]

[1]Chongqing Engineering Laboratory of Environmental Hydraulic Engineering, Chongqing Jiaotong University, Chongqing 400074, China

[2] School of River and Ocean Engineering, Chongqing Jiaotong University, Chongqing 400074, China

✉ Corresponding author, Jianxin Fan (Ph.D, Professor), jxfanw@cqjtu.edu.cn;

Tel: 86-23-62652714; fax: 86-23-62652714.

Content

S1. Reasons for choosing the selected feedstock and pyrolysis temperatures.

S2. Biochar characterization methods.

S3. The results and discussion of the biochar characterization.

Table S1. The pH and variation in DOM quality indices of biochars.

Table S2. Secondary structures in different fouling layers.

Table S3. The experiment group in the soil incubation experiment.

Table S4. The three-way Analysis of Variance (ANOVA) ($p < 0.05$) results for the effects of different treatments on incubated soil (* $p < 0.05$; ** $p < 0.01$, *** $p < 0.001$).

Table S5. Alpha-diversity indices of bacterial communities in soil samples under the different treatments.

Figure S1. Scanning electron microscope (SEM) images of biochar samples.

Figure S2. The FTIR spectra of different biochar samples produced at different pyrolysis temperatures.

Figure S3. The UV–Vis spectra of DOM derived from biochars produced at different pyrolysis temperatures.

Figure S4. The characteristic parameters ($SUVS_{254}$ ($L \cdot mg^{-1} \cdot m^{-1}$)) of the UV–Vis spectra of the DOM released from the biochars produced at different pyrolysis temperatures.

Figure S5. The EEM of DOM derived from biochars produced at different pyrolysis temperatures.

Figure S6. Concentrations of Fe in the soil phase with different treatments during the incubation.

Figure S7. Relative abundances in bacterial community composition among the different amendment treatments at the genus level.

Figure S8. Relative abundances of main AsOB, AsRB, and FeRB among the different amendment treatments.

S1. Reasons for choosing the selected feedstock and pyrolysis temperatures

R. Kushwaha et al. have established that peanut shells are efficient biochar for the removal of arsenic (As) (Li et al., 2021). K. Z. Benis et al. highlighted that unmodified biochar is generally a poor sorbent for As species due to static repulsion between the As oxyanions and the negatively charged biochar surface (Zoroufchi Benis et al., 2020). Accordingly, choosing peanut shells as the feedstock for the unmodified biochar is beneficial for investigating its soil modification abilities that affect the mobility of As.

The yield of biochar decreases with increasing pyrolysis temperature. The yield of peanut shells biochar was 21.9% when the pyrolysis temperature increased to 700°C (Tomczyk et al., 2020). Therefore, we choose 300°C, 400°C, and 500°C as the pyrolysis temperatures to ensure high yields of the biochar.

S2. Biochar characterization methods

The pH was determined at a biochar-to-water ratio of 1:10 and measured with a pH analyzer (PHS-3F, Rex Electric Chemical, China) after half an hour of standing. The biochar morphology was monitored via a scanning electron microscope (SEM, JEOL, Japen) at a 5000 V accelerating voltage and a resolution of 1.4 nm. The functional groups in the samples (using a biochar-to-KBr mass ratio of 1:60) were analyzed by Fourier Transformation Infrared Spectroscopy (Nicolet iS 50, Thermo Scientific, USA), scanning from 450 to 4000 cm^{-1} with 65 scans at a resolution of 4 cm^{-1}.

Dissolved organic matter (DOM) was analyzed using a total organic carbon (TOC) analyzer (TOC-LCPH/CPN, Shimadzu, Japan), and was represented as dissolved organic carbon (DOC). The DOM extraction methods was from Li et al. (Li et al., 2022). A total of 1 g of biochar was taken and added to a centrifuge tube at a ratio of 1:40 (w/v) with Milli-Q water. The tube was placed in a constant temperature shaking bed (200rpm, 25±1°C) in the dark, and shaken for 24 hours. The plastic centrifuge tube was then centrifuged at 4000 r/min for 15 minutes. The filtrate obtained after the mixture was passed through a 0.45 μm filter head was the extracted DOM of the biochar. The obtained filtrate was stored in a refrigerator at 4°C and the sample analysis was completed within 5 days.

UV–visible spectrophotometry (UV–2600, Shimadzu, Japen) was used to measure the UV–visible absorption values of the DOM samples at wavelengths ranging from 200 to 600 nm with a 1 nm interval. The measurements were performed using Milli-Q water as the blank, and the absorbance of the sample was obtained by subtracting the absorbance of the blank water. Specific Ultraviolet Absorbance at 254 nm ($SUVA_{254}$) indicates the molecular weight and aromaticity degree of the DOM (Wang et al., 2013), which was calculated using the formula UV 254/ (A × B), where

UV_{254} is the absorbance of the sample at 254 nm, A is the optical path diameter (0.01 m), and B is the DOM content (mg/L).

A fluorescence spectrophotometer (F-4600, Tokyo, Japan) was used to measure the EEM spectra of the DOM extracted from the different biochars. The scanning range was set from 200 to 550 nm for excitation and from 250 to 650 nm for emission, with excitation and emission slit widths of 5 nm, and a scan speed of 12000 nm/min.

To analyze the biochar characterization results, parallel factor analysis (PARAFAC) was performed using the DOM Fluor v.1.7 Toolbox in MATLAB R2016a (MathWorks, USA) to optimize the EEM data. The spectral parameters Fluorescence Index (FI), Biological Index (BIX), and Humification Index (HIX) were calculated.

S3. The results and discussion of the biochar characterization

The prepared biochars were all alkaline (pH > 7), and the pH increased from 7.82 to 9.87 when the pyrolysis temperature increased from 300 to 500°C (Supplementary Table S2). This trend is consistent with the established literature (Chen et al., 2014; Ding et al., 2014; Tomczyk et al., 2020; Yuan et al., 2011). The elevation in pH values primarily arises from the enhanced pyrolysis temperature, causing the formation of alkali salts from organic substances (Ronsse et al., 2013; Spokas et al., 2012; Zhao et al., 2017).

The SEM images of the three groups of biochar are displayed in Fig. S1. From the images, it can be observed that the surfaces of all three types of biochar were relatively rough and contained abundant pore structures. Notably, BC500 exhibited a more pronounced porous structure compared to BC300 and BC400, with its surface appearing even rougher. This suggested that variations in pyrolysis temperature can indeed influence both the pore structure and surface morphology of the biochar.

The FTIR spectra of the biochars produced at different pyrolysis temperatures are depicted in Fig. S2. BC300, BC400, and BC500 exhibited similar absorption peaks, primarily attributed to characteristic functional groups such as -COOH (1550~1650 cm^{-1}), C=O (~1100 cm^{-1}), and C=C (~800 cm^{-1}). However, it could also be observed that with the increase in pyrolysis temperature, certain functional groups gradually disappeared. For instance, in BC500, there was an absence of absorption peaks associated with phenolic and alcoholic hydroxyl groups (3400~3500 cm^{-1}), as well as saturated aliphatic C-H bonds (2800~2900 cm^{-1}).

The DOC content of the biochar samples decreased with increasing pyrolysis temperature (Supplementary Table S2). This might be caused by the decomposition of organic matter at higher

temperatures (Tomczyk et al., 2020). The decrease in DOC content may be attributed to the reduction in the violate substance content and the increase in the fixed carbon content (Liu et al., 2015). Moreover, the pyrolysis products obtained at a low temperature may undergo secondary actions such as decomposition, condensation, and polymerization, leading to the formation of stable poly-aromatic hydrocarbons and a decrease in the DOC content of the biochar (Zhang et al., 2020).

From Table S2, it is evident that the FI values of the peanut shell biochar decreased with increasing pyrolysis temperature, all of which were less than 1.2. This suggests that the DOM in all three biochar samples originated primarily from soil-derived DOM, with an increase in the aromatic substance content with rising pyrolysis temperature. As the pyrolysis temperature gradually increased, the BIX progressively decreased, indicating a reduction in the proportion of protein-like substances and their biological availability in the biochar-derived DOM. Meanwhile, the HIX decreased with increasing pyrolysis temperature, signifying that at higher pyrolysis temperatures, the DOM derived from the biochar exhibited higher humification and aromatization, resulting in a more complex structure.

The effects of different pyrolysis temperatures on the UV–Vis spectrum of the biochar-derived DOM are shown in Fig. S3. Within the wavelength range of 200-400 nm, the absorption coefficient exponentially decreased, and then stabilized. This phenomenon may be associated with the transformation of aromatic substances in the biochar (Gui et al., 2020). Fig. S4 illustrates the $SUVA_{254}$ values for the various biochars. It is evident that the $SUVA_{254}$ values of the biochar-derived DOM gradually increased with rising pyrolysis temperature, indicating a higher aromaticity and molecular weight at higher temperatures. The $SUVA_{254}$ values for all three biochar samples were less than $3\mathrm{L} \cdot \mathrm{mg}^{-1} \cdot \mathrm{m}^{-1}$, indicating a prevalence of hydrophilic components (Tang et al., 2016).

It can be inferred that BC300 contained fulvic and humic acids in the visible light region. BC400 contained both fulvic acid in the UV region and fulvic acid in the visible light region. BC500 contained fulvic acid in the UV region (Supplementary Fig. S5 and Table S3).

Supplementary Table S1. The pH and variation in DOM quality indices of biochars.

Sample	pH	DOC (mg/L)	FI	BIX	HIX	Ash
BC300	7.82	221.8	0.9860	1.123	0.7580	19.89
BC400	8.94	69.25	0.8951	1.006	0.8297	25.92
BC500	9.87	9.578	0.7043	0.7790	0.8866	28.06

The Fluorescence Index (FI) was defined as the ratio of the fluorescence intensity at 470 nm and 520 nm with an excitation wavelength of 370 nm (Gui et al., 2020). The Humification Index (HIX) was calculated as the ratio of the area of the emission intensity between 435 and 480 nm to the area of the emission intensity between 300 and 345 nm at an excitation wavelength of 254 nm (Wu et al., 2019). The Biological Index (BIX), which is an indicator of freshly produced DOM, is the ratio of the emission intensities at 380–430 nm at 310 nm (Ex) (Lee et al., 2018).

Supplementary Table S2. Characteristic peaks for substances in DOM fluorescence spectra (Bai et al., 2020; Huo et al., 2023; Wen et al., 2023).

E_X/E_M (nm)	Representative substance
210~230, 260~280/280~310	Tyrosine-like substance
220~240, 260~280/320~350	Tryptophan-like substance
240~260/380~410	UV-zone analog of fulvic acid-like substances
330~350/380~410	Visible-zone analog of fulvic acid-like substances
260~300/475~510	Humic acid-like substance

Supplementary Table S3. The experiment group in the soil incubation experiment.

Sample	Biochar application rate (w: w)	Initial soil pH
CK-P1	Equivalent deionized water	5.5
CK-P2	Equivalent deionized water	7.0
CK-P3	Equivalent deionized water	8.5
SBC300-P1	5% BC300	5.5
SBC300-P2	5% BC300	7.0
SBC300-P3	5% BC300	8.5
SBC400-P1	5% BC400	5.5
SBC400-P2	5% BC400	7.0
SBC400-P3	5% BC400	8.5
SBC500-P1	5% BC500	5.5
SBC500-P2	5% BC500	7.0
SBC500-P3	5% BC500	8.5

CK means no biochar; SBC300, SBC400, and SBC500 represent the soils with the application of different biochars (BC300, BC400, and BC500, respectively). Application rates refer to the ratio of amendment mass-to-soil mass (w/w). BC300, BC400, and BC500 represent the biochars prepared from peanut shells under different pyrolysis temperatures (300°C, 400°C, and 500°C, respectively).

Supplementary Table S4. The three-way Analysis of Variance (ANOVA) ($p < 0.05$) results for the effects of different treatments on incubated soil (* $p < 0.05$; ** $p < 0.01$, *** $p < 0.001$).

Independent Variable	P value of Dependent Variable									
	pH	Eh	DOC	Fe (II)	TFe	HCL-Fe	As (III)	TAs	A-As	Amo-Fe-As
Biochar	< 2e-16 ***	< 2e-16 ***	<2e-16 ***	2.02e-12 ***	<2e-16 ***	< 2e-16 ***	<2e-16 ***	< 2e-16 ***	< 2e-16 ***	0.00314 **
Soil pH	< 2e-16 ***	0.614	0.5320	0.6262	0.2145	0.1832	0.885	0.168534	0.2458	0.01671 *
Time	< 2e-16 ***	< 2e-16 ***	<2e-16 ***	< 2e-16 ***	<2e-16 ***	< 2e-16 ***	<2e-16 ***	< 2e-16 ***	5.87e-07 ***	2.43e-09 ***
Biochar + soil pH	8.18e-05 ***	6.49e-14 ***	0.7759	0.1064	0.0018 **	0.0084 **	0.930	0.321722	0.4204	0.87736
Biochar + time	< 2e-16 ***	< 2e-16 ***	0.0033 **	3.58e-08 ***	<2e-16 ***	< 2e-16 ***	<2e-16 ***	< 2e-16 ***	0.0214 *	2.28e-15 ***
Soil pH + time	7.10e-13 ***	4.84e-16 ***	0.1117	0.9888	0.6931	0.1142	0.928	0.166674	0.7294	0.10078
Biochar + soil pH + time	5.70e-10 ***	< 2e-16 ***	0.2238	0.0261 *	0.0262 *	1.68e-05 ***	0.958	0.000738 ***	0.1842	0.00439 **

The independent variables biochar, soil pH, and time indicate the application of different biochars (BC300, BC400, BC500), the initial soil pH (5.5, 7.0, 8.5), and the incubation day (1st, 2nd, 4th, 7th, 15th, 21st), respectively. The biochar was the main independent factor.

Supplementary Table S5. Alpha-diversity indices of bacterial communities in soil samples under the different treatments.

Experiment Group	Day	OTUs	Species richness		Species diversity	
			ACE	Chao1	Simpson	Shannon
Original Soil	\	42753	4692.141	4650.634	0.003418	6.775241
CK-P1	4th	44314	4411.889	4375.053	0.004783	6.554813
	21st	33966	4424.877	4373.148	0.003483	6.69907
CK-P2	4th	44423	4446.192	4491.758	0.004539	6.593112
	21st	36477	4710.468	4666.779	0.003689	6.667046
CK-P3	4th	32871	3349.172	3321.381	0.004352	6.557445
	21st	45755	4575.193	4513.108	0.00593	6.520869
BC300-P1	4th	47977	4271.431	4252.012	0.005103	6.502218
	21st	40654	4733.55	4706.451	0.003395	6.745679
BC300-P2	4th	45023	4367.149	4292.353	0.007019	6.403359
	21st	40039	4393.25	4344	0.003662	6.676 957
BC300-P3	4th	41056	3847.061	3810.542	0.00917	6.141673
	21st	45340	4749.583	4762.062	0.003382	6.749612
BC400-P1	4th	44214	3884.495	3891.586	0.011072	5.972336
	21st	47211	4680.253	4719.247	0.00459	6.665632
BC400-P2	4th	41346	3952.413	3923.392	0.010688	6.082036
	21st	43661	4642.148	4612.304	0.003496	6.718768
BC400-P3	4th	47523	4382.306	4430.66	0.005754	6.451578
	21st	44511	4674.054	4682.101	0.003466	6.746189
BC500-P1	4th	48342	4448.781	4518.911	0.01078	6.204063
	21st	46334	4847.824	4825.061	0.002845	6.843013
BC500-P2	4th	59838	4808.578	4842.723	0.007318	6.451542
	21st	47446	4884.877	4881.36	0.002787	6.847462
BC500-P3	4th	43361	4485.754	4451.816	0.005357	6.558787
	21st	43539	4945.874	4937.875	0.004211	6.678844

Treatment abbreviations are defined in Table S3.

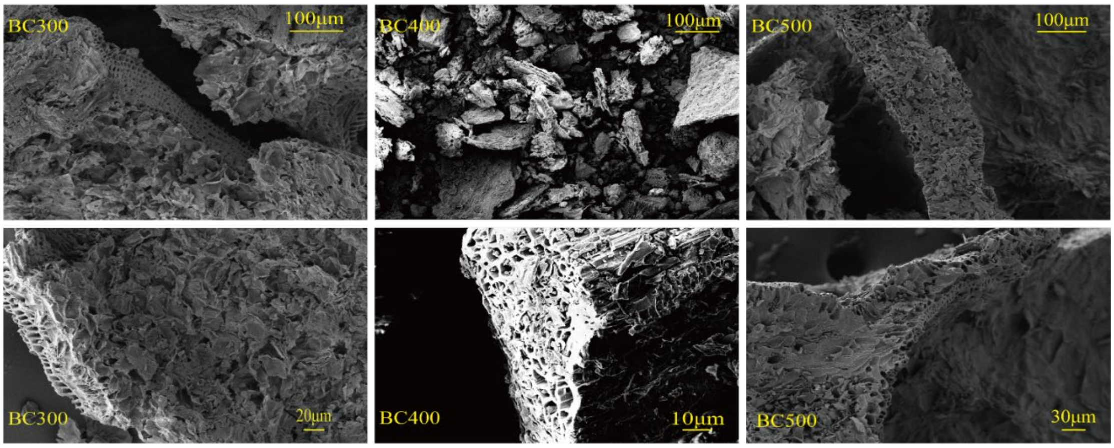

Supplementary Figure S1. Scanning electron microscopy (SEM) images of biochar samples.

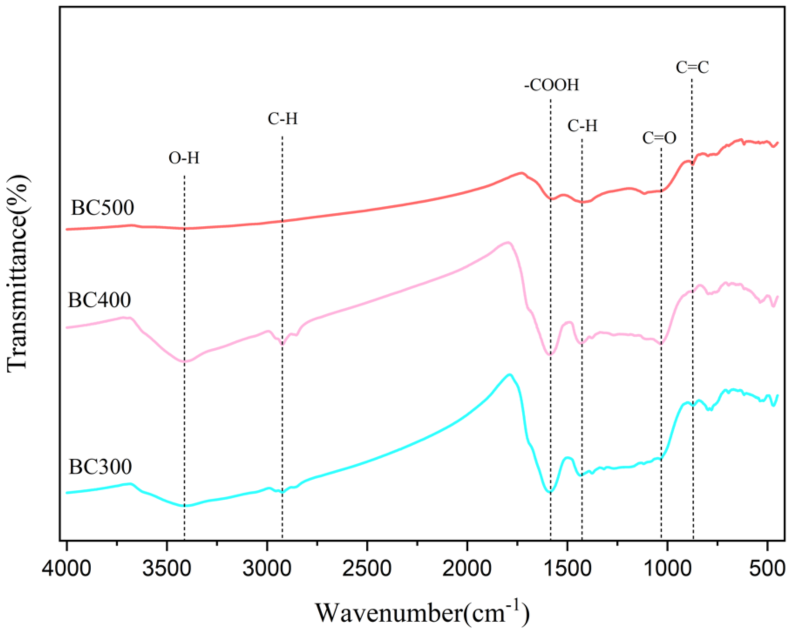

Supplementary Figure S2. The FTIR spectra of different biochar samples produced at different pyrolysis temperatures.

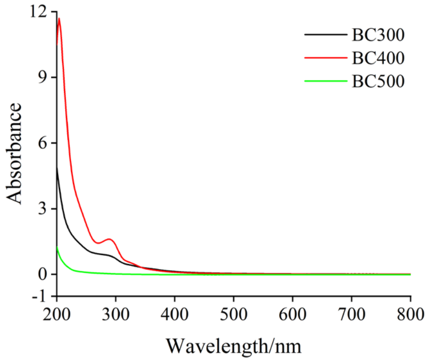

Supplementary Figure S3. The UV–Vis spectra of DOM derived from biochars produced at different pyrolysis temperatures.

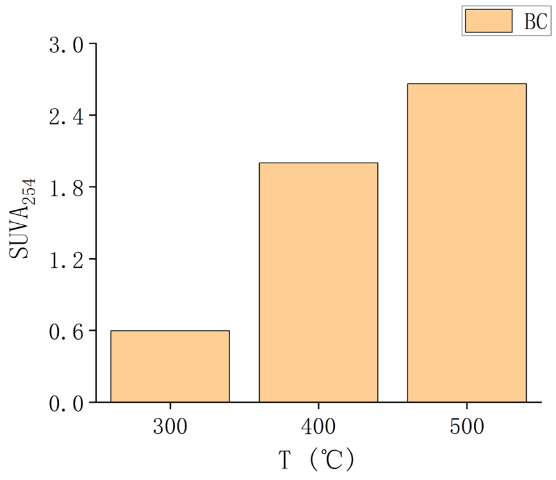

Supplementary Figure S4. The characteristic parameters (SUVS254 (L·mg^{-1}·m^{-1})) of the UV–Vis spectra of the DOM released from the biochars produced at different pyrolysis temperatures.

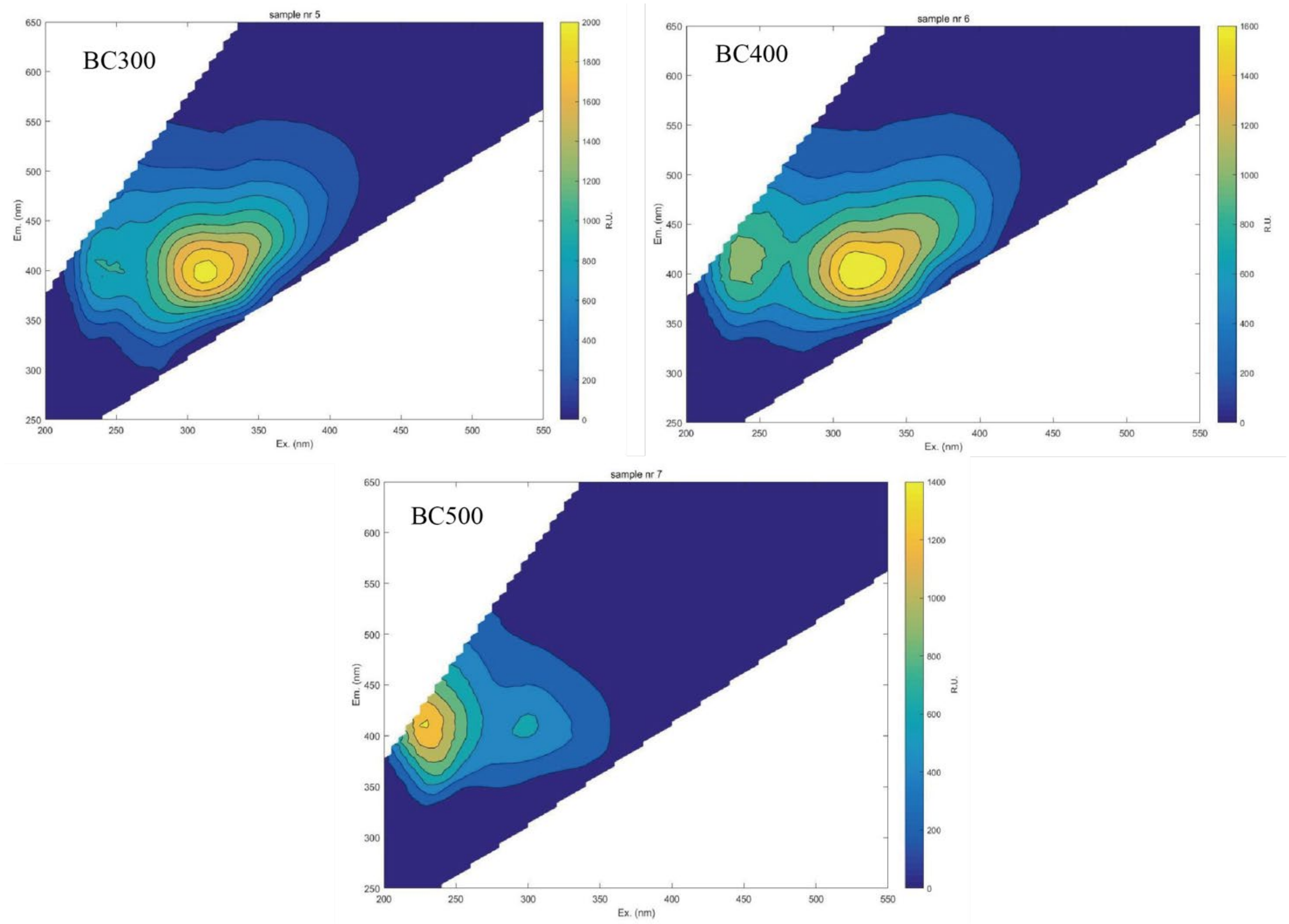

Supplementary Figure S5. The EEM of DOM derived from biochars produced at different pyrolysis temperatures.

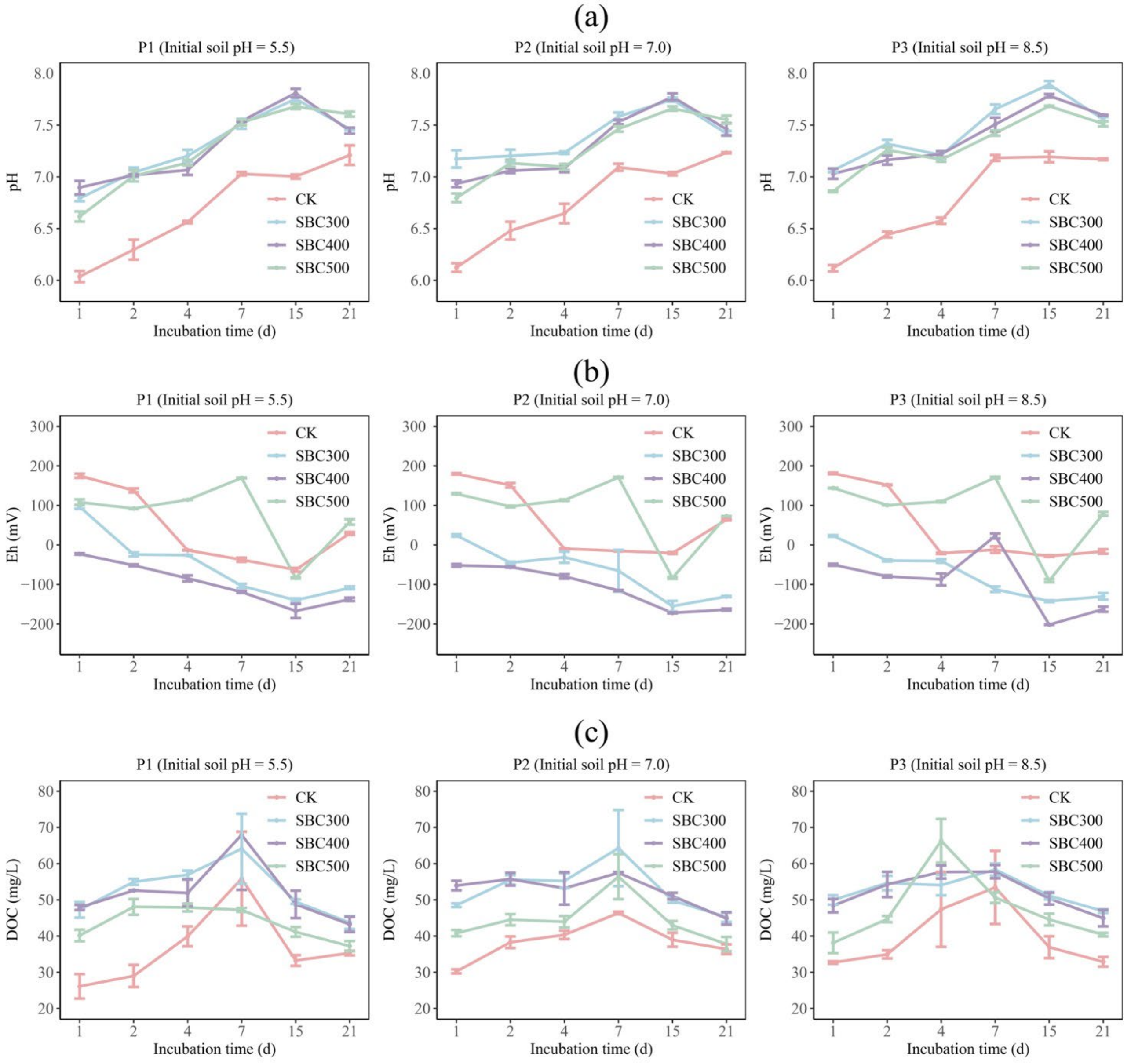

Supplementary Figure S6. Variation in (a) pH, (b) Eh, and (3) concentration of DOC in the supernatant with different treatments during the incubation. Values are the mean ± standard error of three replicates. Different letters in the same day indicate significant differences among treatments ($p < 0.05$). Treatment abbreviations are defined in Table S3.

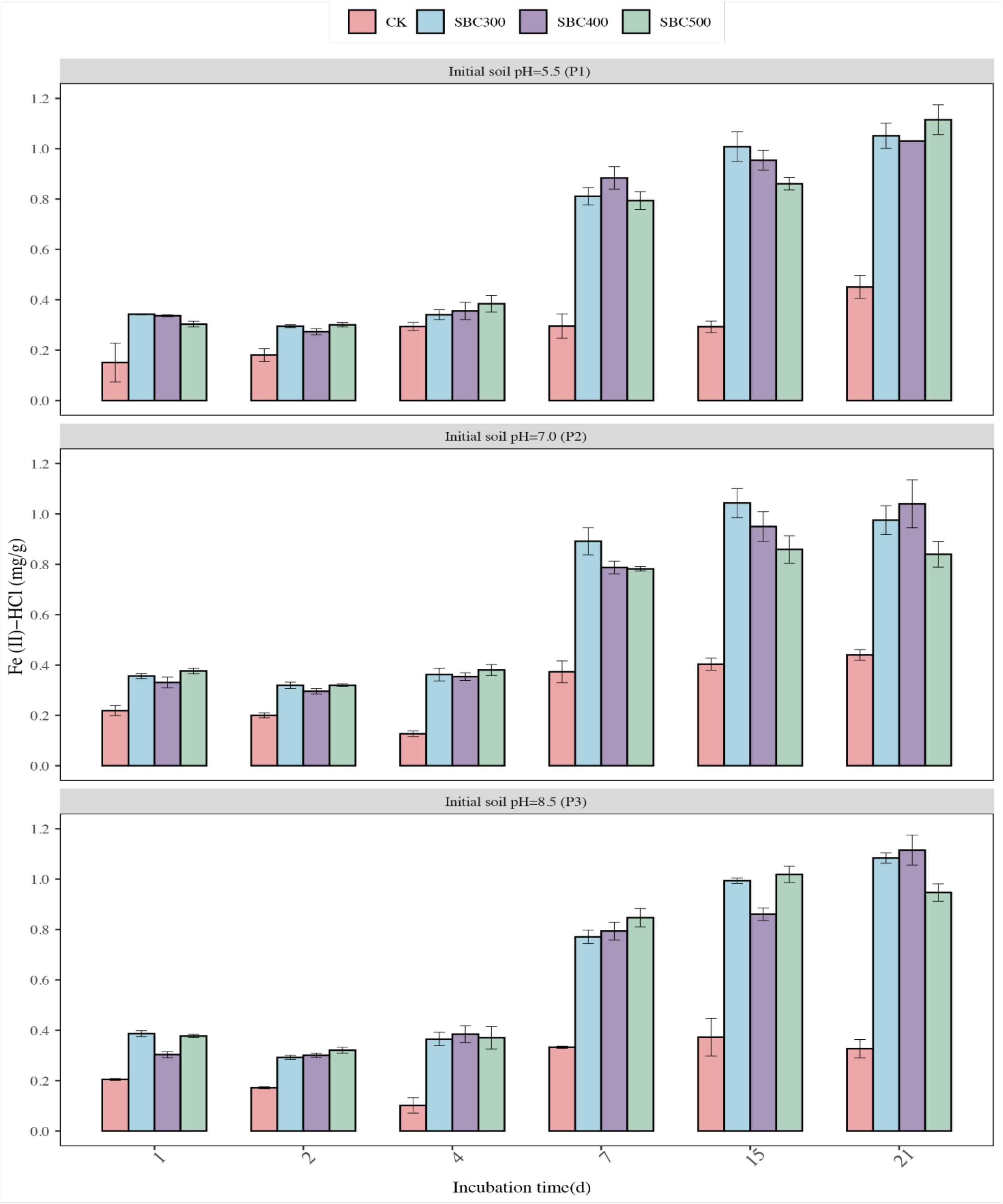

Supplementary Figure S7. Concentrations of Fe in the soil phase with different treatments during the incubation. Values are the mean ± standard error of three replicates. Treatment abbreviations are defined in Table S3.

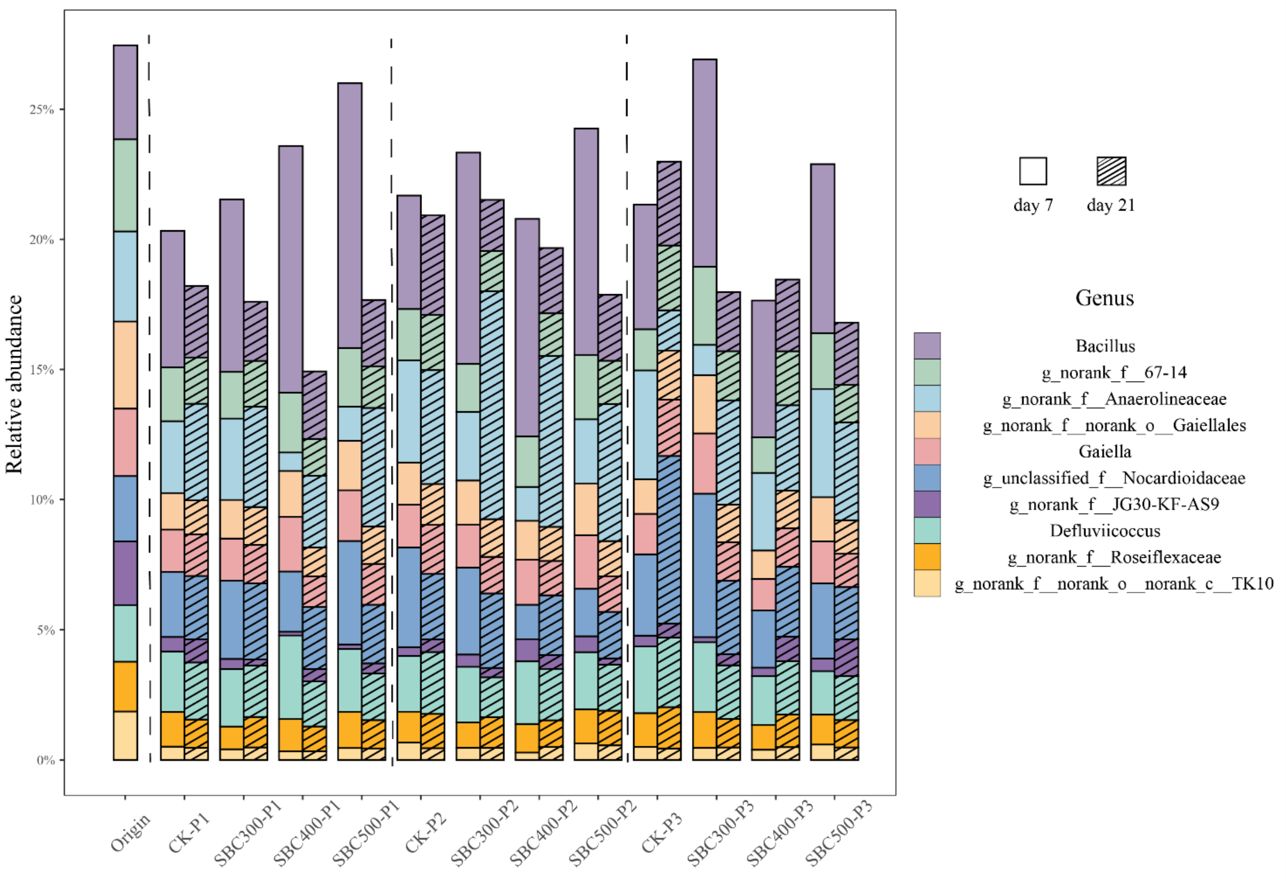

Supplementary Figure S8. Relative abundances in bacterial community composition among the different amendment treatments at the genus level. The bar color represents the bacterial phylum. Origin indicated the fresh soil without any treatment. Treatment abbreviations are defined in Table S3.

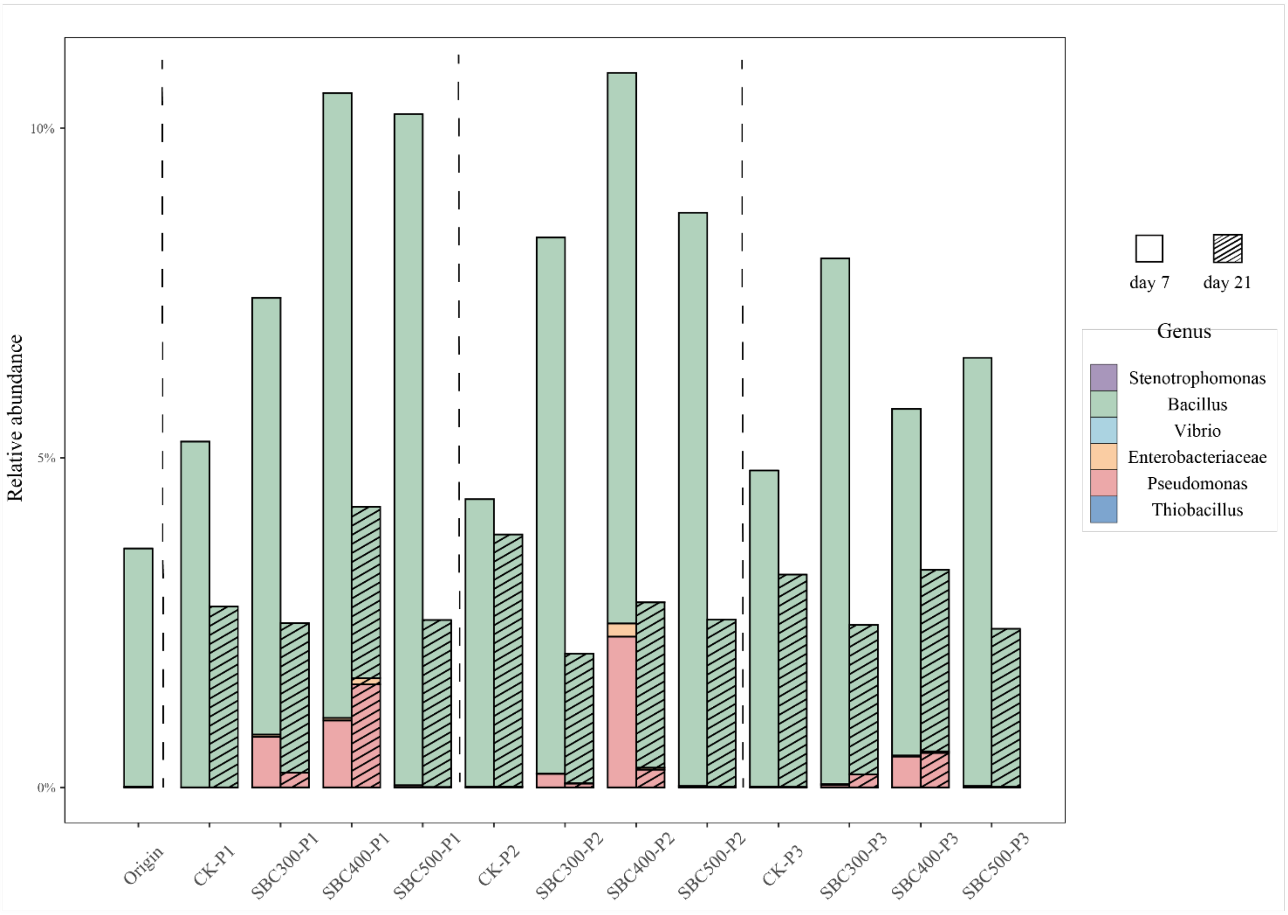

Supplementary Figure A9. Relative abundances of main AsOB, AsRB, and FeRB among the different amendment treatments. The bar color represents the bacterial phylum. Origin indicates the fresh soil without any treatment. Treatment abbreviations are defined in Table S3.

References

Bai, Y., Ding, A., Zhang, S., 2020. Characterizing chromophoric dissolved organic matter in Guanting Reservoir Beijing using excitation-emission matrix fluorescence and parallel factor analysis (No. EGU2020-17510). Presented at the EGU2020, Copernicus Meetings. https://doi.org/10.5194/egusphere-egu2020-17510

Chen, T., Zhang, Yaxin, Wang, H., Lu, W., Zhou, Z., Zhang, Yuancheng, Ren, L., 2014. Influence of pyrolysis temperature on characteristics and heavy metal adsorptive performance of biochar derived from municipal sewage sludge. Bioresource Technology 164, 47–54. https://doi.org/10.1016/j.biortech.2014.04.048

Ding, W., Dong, X., Ime, I.M., Gao, B., Ma, L.Q., 2014. Pyrolytic temperatures impact lead sorption mechanisms by bagasse biochars. Chemosphere 105, 68–74. https://doi.org/10.1016/j.chemosphere.2013.12.042

Gui, X., Liu, C., Li, F., Wang, J., 2020. Effect of pyrolysis temperature on the composition of DOM in manure-derived biochar. Ecotoxicology and Environmental Safety 197, 110597. https://doi.org/10.1016/j.ecoenv.2020.110597

Huo, P., Zhang, W., Jia, H., Chen, J., Gao, P., 2023. Characteristics of optical properties of DOM and nutrients in rainwater of different ecological areas of a large reservoir in China. Chemosphere 342, 140091. https://doi.org/10.1016/j.chemosphere.2023.140091

Lee, M.-H., Ok, Y.S., Hur, J., 2018. Dynamic variations in dissolved organic matter and the precursors of disinfection by-products leached from biochars: Leaching experiments simulating intermittent rain events. Environmental Pollution 242, 1912–1920. https://doi.org/10.1016/j.envpol.2018.07.073

Li, J., Zhang, Y., Wang, F., Wang, L., Liu, J., Hashimoto, Y., Hosomi, M., 2021. Arsenic immobilization and removal in contaminated soil using zero-valent iron or magnetic biochar amendment followed by dry magnetic separation. Science of The Total Environment 768, 144521. https://doi.org/10.1016/j.scitotenv.2020.144521

Li, L.-P., Liu, Y.-H., Ren, D., Wang, J.-J., 2022. Characteristics and chlorine reactivity of biochar-derived dissolved organic matter: Effects of feedstock type and pyrolysis temperature. Water Research 211, 118044. https://doi.org/10.1016/j.watres.2022.118044

Liu, P., Ptacek, C.J., Blowes, D.W., Berti, W.R., Landis, R.C., 2015. Aqueous Leaching of Organic Acids and Dissolved Organic Carbon from Various Biochars Prepared at Different Temperatures. J. Environ. Qual. 44, 684–695. https://doi.org/10.2134/jeq2014.08.0341

Ronsse, F., van Hecke, S., Dickinson, D., Prins, W., 2013. Production and characterization of slow pyrolysis biochar: influence of feedstock type and pyrolysis conditions. GCB Bioenergy 5, 104–115. https://doi.org/10.1111/gcbb.12018

Spokas, K.A., Cantrell, K.B., Novak, J.M., Archer, D.W., Ippolito, J.A., Collins, H.P., Boateng, A.A., Lima, I.M., Lamb, M.C., McAloon, A.J., Lentz, R.D., Nichols, K.A., 2012. Biochar: A Synthesis of Its Agronomic Impact beyond Carbon Sequestration. Journal of Environmental Quality 41, 973–989. https://doi.org/10.2134/jeq2011.0069

Tang, J., Li, X., Luo, Y., Li, G., Khan, S., 2016. Spectroscopic characterization of dissolved organic matter derived from different biochars and their polycylic aromatic hydrocarbons (PAHs) binding affinity. Chemosphere 152, 399–406. https://doi.org/10.1016/j.chemosphere.2016.03.016

Tomczyk, A., Sokołowska, Z., Boguta, P., 2020. Biochar physicochemical properties: pyrolysis temperature and feedstock kind effects. Rev Environ Sci Biotechnol 19, 191–215. https://doi.org/10.1007/s11157-020-09523-3

Wang, Y., Zhang, D., Shen, Z., Feng, C., Chen, J., 2013. Revealing Sources and Distribution Changes of Dissolved Organic Matter (DOM) in Pore Water of Sediment from the Yangtze Estuary. PLoS One 8, e76633. https://doi.org/10.1371/journal.pone.0076633

Wen, Y., Xiao, M., Chen, Z., Zhang, W., Yue, F., 2023. Seasonal Variations of Dissolved Organic Matter in Urban

Rivers of Northern China. Land 12, 273. https://doi.org/10.3390/land12020273
Wu, H., Qi, Y., Dong, L., Zhao, X., Liu, H., 2019. Revealing the impact of pyrolysis temperature on dissolved organic matter released from the biochar prepared from Typha orientalis. Chemosphere 228, 264–270. https://doi.org/10.1016/j.chemosphere.2019.04.143
Yuan, J.-H., Xu, R.-K., Zhang, H., 2011. The forms of alkalis in the biochar produced from crop residues at different temperatures. Bioresource Technology 102, 3488–3497. https://doi.org/10.1016/j.biortech.2010.11.018
Zhang, X., Zhang, P., Yuan, X., Li, Y., Han, L., 2020. Effect of pyrolysis temperature and correlation analysis on the yield and physicochemical properties of crop residue biochar. Bioresource Technology 296, 122318. https://doi.org/10.1016/j.biortech.2019.122318
Zhao, S.-X., Ta, N., Wang, X.-D., 2017. Effect of Temperature on the Structural and Physicochemical Properties of Biochar with Apple Tree Branches as Feedstock Material. Energies 10, 1293. https://doi.org/10.3390/en10091293
Zoroufchi Benis, K., Motalebi Damuchali, A., Soltan, J., McPhedran, K.N., 2020. Treatment of aqueous arsenic – A review of biochar modification methods. Science of The Total Environment 739, 139750. https://doi.org/10.1016/j.scitotenv.2020.139750
